# Supplementary material for: Actomyosin drives cancer cell nuclear dysmorphia and threatens genome stability
Source: Nat Commun. 2017 Jul 24;8:16013. doi: 10.1038/ncomms16013 (PMC5527285; doi:10.1038/ncomms16013)
Supplement: Supplementary Information [file ncomms16013-s1.pdf]

Title of file for HTML: Supplementary Information
Description: Supplementary Figures and Supplementary Tables

Title of file for HTML: Supplementary Movie 1
Description: Nuclear morphology and dynamics in nontargeting siRNA-transfected cells. HeLa Kyoto cells expressing AcGFPLAP2ß (green) and H2B-mCherry (magenta) were transfected with nontargeting control siRNA and imaged every 3 min starting 50 h after transfection.

Title of file for HTML: Supplementary Movie 2
Description: Nuclear morphology and dynamics in PPP1R12A-depleted cells. HeLa Kyoto cells expressing AcGFP-LAP2ß (green) and H2B-mCherry (magenta) were transfected with PPP1R12A siRNA and imaged every 3 min starting 50 h after transfection.

Title of file for HTML: Supplementary Movie 3
Description: 3D-reconstructed nuclei of non-targeting siRNA transfected cells. HeLa Kyoto cells were transfected with nontargeting control siRNA and fixed 56 h after transfection. Cells were stained with anti-lamin B1 antibodies (green) and DAPI (red).

Title of file for HTML: Supplementary Movie 4
Description: Z-scan through 3D-reconstructed nuclei of non-targeting siRNA transfected cells. HeLa Kyoto cells were transfected with non-targeting control siRNA and fixed 56 h after transfection. Cells were stained with anti-lamin B1 antibodies (green) and DAPI (red).

Title of file for HTML: Supplementary Movie 5
Description: 3D-reconstructed nuclei of PPP1R12A-depleted cells. HeLa Kyoto cells were transfected with PPP1R12A siRNA and fixed 56 h after transfection. Cells were stained with anti-lamin B1 antibodies (green) and DAPI (red).

Title of file for HTML: Supplementary Movie 6
Description: Nuclear compartment integrity assay in nontargeting siRNA-transfected cells. HeLa Kyoto cells expressing AcGFPNES (green) and mCherry-NLS (magenta) were transfected with nontargeting control siRNA and imaged every 2 min starting 40 h after transfection.

Title of file for HTML: Supplementary Movie 7
Description: Nuclear compartment integrity assay in PPP1R12A-depleted cells. HeLa Kyoto cells expressing AcGFP-NES (green) and mCherry-NLS (magenta) were transfected with PPP1R12A siRNA and imaged every 2 min starting 40 h after transfection.

Title of file for HTML: Supplementary Movie 8
Description: Mitosis in non-targeting siRNA-transfected cells. HeLa Kyoto cells expressing AcGFP-LAP2ß (green) and H2B-mCherry (magenta) were transfected with non-targeting control siRNA and imaged every 3 min starting 50 h after transfection.

Title of file for HTML: Supplementary Movie 9
Description: Mitosis in PPP1R12A-depleted cells. HeLa Kyoto cells expressing AcGFP-LAP2ß (green) and H2B-mCherry (magenta) were transfected with PPP1R12A siRNA and imaged every 3 min starting 50 h after transfection.

Title of file for HTML: Supplementary Movie 10
Description: Intravital imaging of nuclear deformation in xenograft tumor. Frames of transplanted MDA-MB-231 cells expressing AcGFP-LAP2ß (green) and H2B-mCherry (magenta) were acquired every 1 min.

Title of file for HTML: Supplementary Movie 11
Description: Intravital imaging of nuclear dynamics in untreated xenograft tumor. Frames of transplanted MDA-MB-231 cells expressing AcGFP-LAP2ß (green) and H2B-mCherry (not shown) were acquired every 1 min.

Title of file for HTML: Supplementary Movie 12
Description: Intravital imaging of nuclear dynamics in Y- 27632-treated xenograft tumor. Frames of transplanted MDA-MB-231 cells expressing AcGFP-LAP2ß (green) and H2B-mCherry (not shown) were acquired every 1 min.

Title of file for HTML: Supplementary Data 1
Description: Phosphatase siRNA screen. The table lists the siRNA catalogue number (Dharmacon), gene symbol and accession number (NCBI) of the targeted mRNA. Furthermore, it lists the quantification of cells with abnormal nuclear morphology for each siRNA pool as derived from the primary screen. These data form the bases for the graph shown in Supplementary Fig. 1a.

Title of file for HTML: Peer Review File
Description:

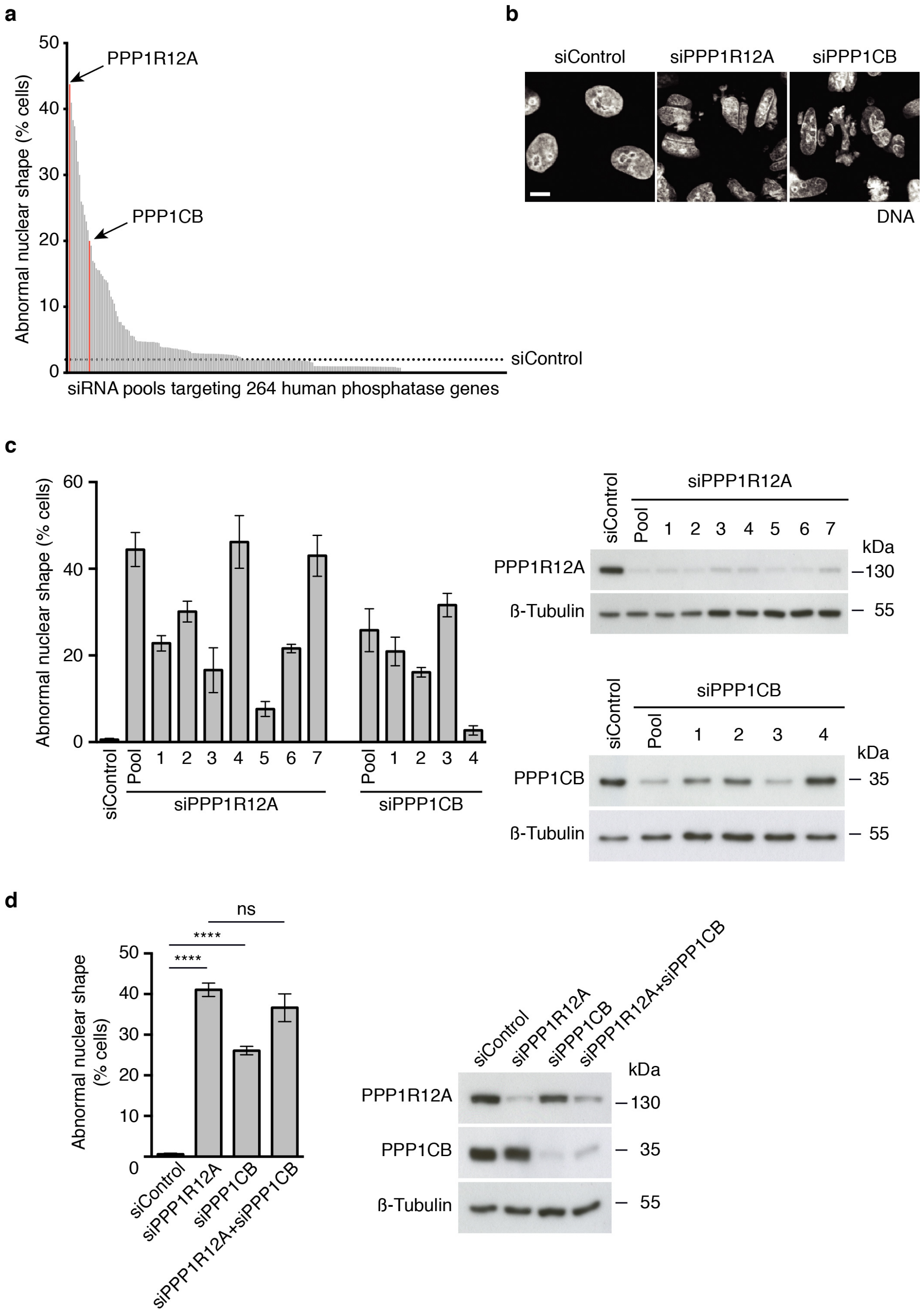

Supplementary Figure 1 | Nuclear phenotype of PPP1R12A and PPP1CB depletion. **(a)** HeLa Kyoto cells were transfected with an siRNA library targeting 264 human phosphatases (Supplemental Data 1). Cells were fixed 72 h after transfection. Cells were stained with anti-α-tubulin antibodies and DAPI. Abnormal nuclear shape was scored visually (Supplemental Data 1). **(b)** Representative DAPI-stained images of cells transfected with PPP1R12A and PPP1CB siRNA duplexes. Scale bar, 10 µm. (c and d) HeLa Kyoto cells were transfected with the indicated siRNA duplexes. Cells were fixed for microscopy or processed for immunoblot analysis 56 h after transfection. **(c)** Nuclear shape quantification (left panel) and immunoblot analysis (right panel) of transfected cells. Error bars, s.d. of three independent experiments (n > 200 cells each). **(d)** Cells transfected with siControl, siPPP1R12A, siPPP1CB or a combination of siPPP1R12A and siPPP1CB were analyzed as in (c). Error bars, s.d. of three independent experiments (n > 200 cells each). Graph shows ****P < 0.0001, ns non-significant (one-way ANOVA Tukey's multiple comparison test).

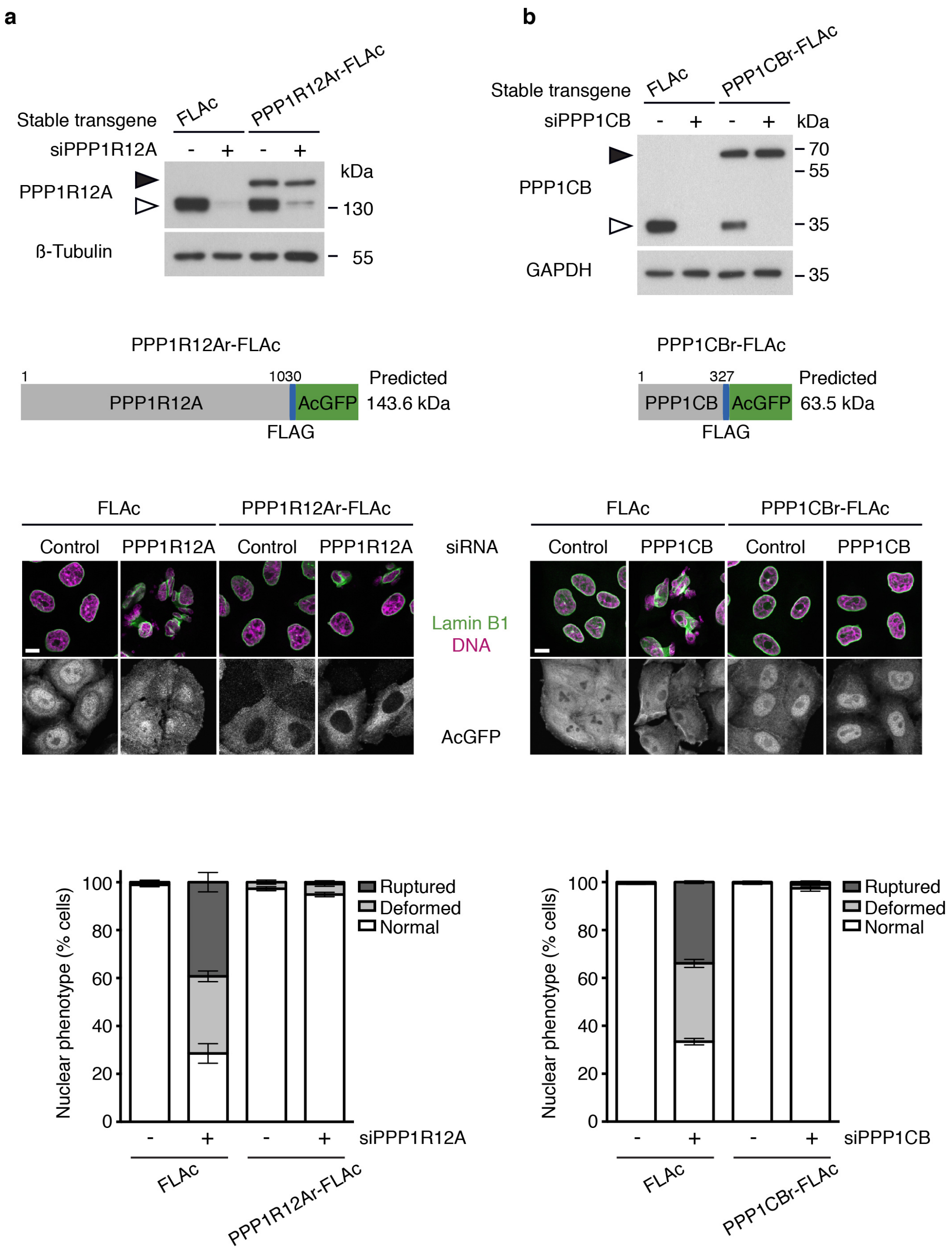

Supplementary Figure 2 | Transgenic rescue experiments of PPP1R12A and PPP1CB depletion. **(a)** Complementation of PPP1R12A depletion with an siRNA resistant PPP1R12Ar-FLAc transgene. **(b)** Complementation of PPP1CB depletion with an siRNA resistant PPP1CBr-FLAc transgene. Immunoblot analysis of cells stably expressing the FLAc tag only, PPP1R12Ar-FLAc or PPP1CBr-FLAc transgene (upper panels). Protein extracts were prepared 56 h after transfection. Schematic illustration of the transgene constructs (middle upper panels). Representative images of stable cell lines expressing the indicated transgenes and transfected with the indicated siRNA duplexes (lower middle panels). Cells were fixed and stained with DAPI and antibodies directed against lamin B1 and AcGFP 56 h after transfection. Quantification of nuclear phenotypes in transgene-positive cells (bottom panels). Scale bar, 10 µm. Error bars, s.d. of three independent experiments (n > 200 cells each).

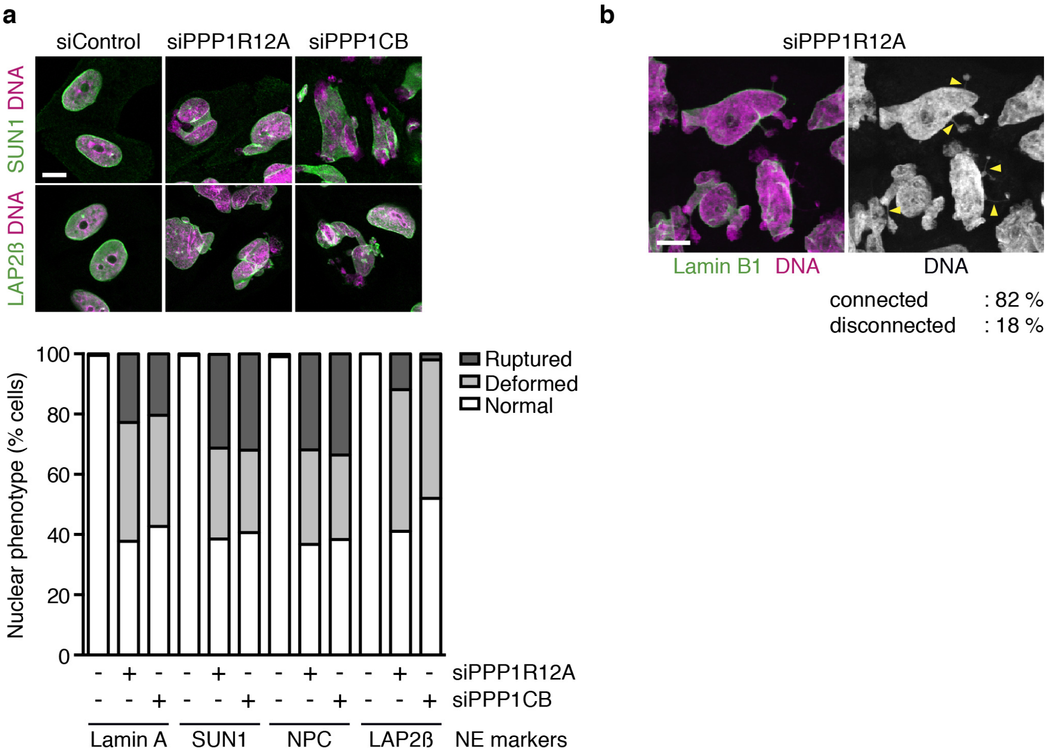

Supplementary Figure 3 | Analysis of LAP2ß, SUN1 and NPC nuclear envelope markers. (a) Representative images of siControl, siPPP1R12A or siPPP1CB-transfected cells (upper panel). Cells were fixed 56 h after transfection and stained with antibodies directed against the indicated nuclear envelope (NE) proteins. Quantification of nuclear phenotype (lower panel) (n > 200 cells each). **(b)** Representative image of siPPP1R12A-transfected cells. Arrowheads indicate DNA bridges connecting chromatin segments to main nuclear body. The percentage of extruded DNA segments in PPP1R12A-depleted cells that are connected or disconnected from the main nuclear body is shown (n > 200 cells each). Scale bars, 10 µm.

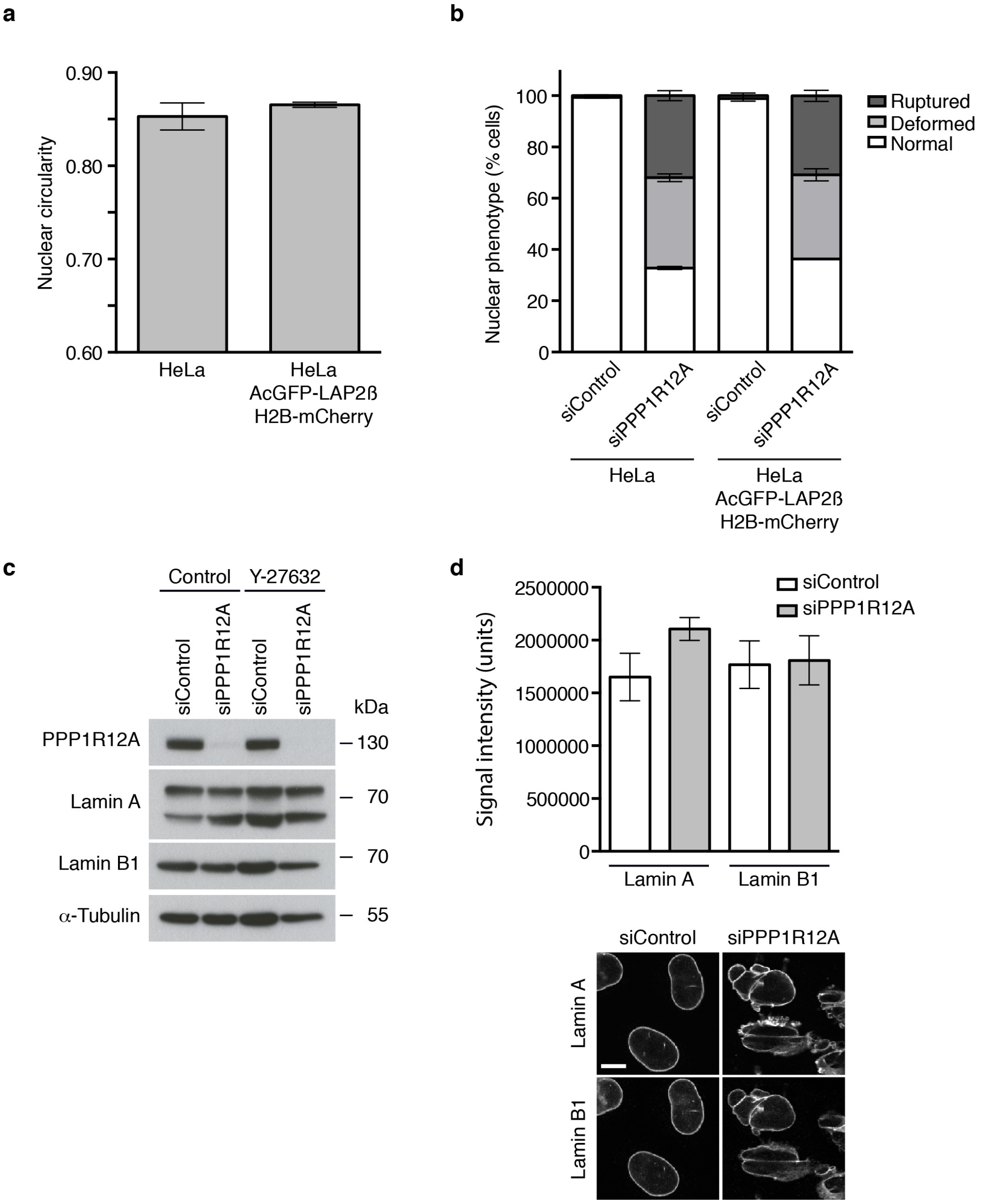

Supplementary Figure 4 | Effect of marker transgene expression on nuclear phenotype and of PPP1R12A depletion on lamin levels. (a) Nuclear circularity of HeLa Kyoto cells and HeLa Kyoto cells stably expressing AcGFP-LAP2ß and H2B-mCherry. (b to d) Cells transfected with control or PPP1R12A siRNA were fixed or harvested 56 h after transfection. Error bars, s.d. of three independent experiments (n > 200 cells each) **(b)** Comparison of nuclear phenotype of PPP1R12A depletion in HeLa Kyoto cells and HeLa Kyoto cells stably expressing AcGFP-LAP2ß and H2B-mCherry. **(c)** Immunoblot analysis of lamin A and lamin B1 levels in PPP1R12A siRNA-transfected cells treated with DMSO or with 5 µM Y-27632. **(d)** Quantification of lamin A and lamin B1 signals in control and PPP1R12A siRNA-transfected cells by immunofluorescence microscopy (top panel). Representative images of cells stained with lamin A and lamin B1 (bottom panel). Error bars, s.d. of three independent experiments (n > 200 cells each) (b and d). Scale bar, 10 µm.

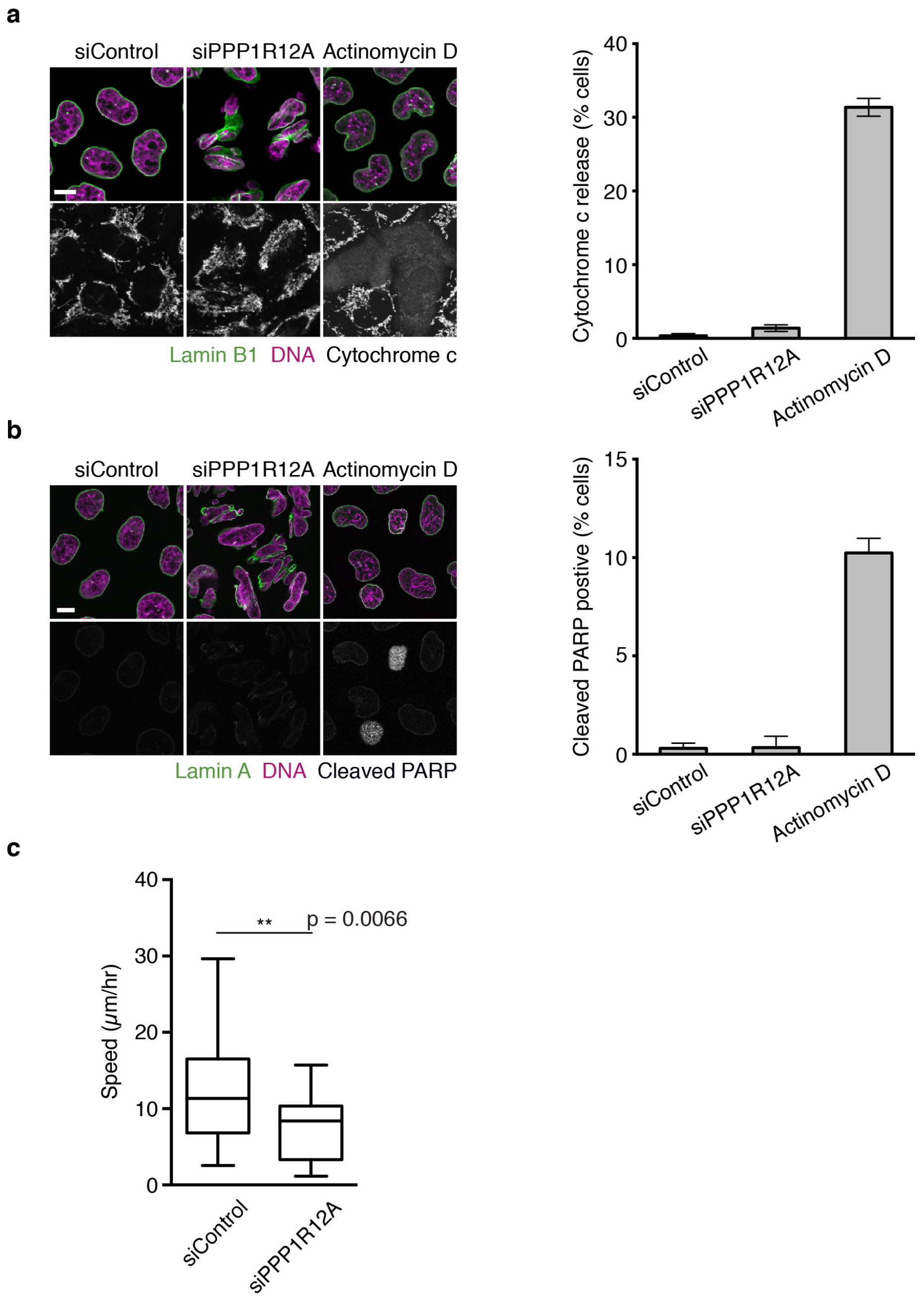

Supplementary Figure 5 | Apoptotic markers and cell migration speed. (a and b) Cells were transfected with the indicated siRNA duplexes and treated with actinomycin D as indicated. **(a)** Representative images of cytochrome c, lamin B1 and DNA (left panel). Quantification of % cells with mitochondrial cytochrome c release (right panel). Error bars, s.d. of three independent experiments (n > 200 cells each). **(b)** Representative images of cleaved PARP, lamin B1 and DNA (left panel). Quantification of % cells with cleaved PARP signal (right panel). Error bars, s.d. of three independent experiments (n > 200 cells each). Scale bars, 10 μm. **(c)** Box and whisker plot shows the cell speed in a 2D cell migration assay of control and PPP1R12A siRNA-transfected MDA-MB-231 cells. Median, quartiles, and extremes are shown, n > 20 cells per condition. Graph shows P value of unpaired t test.

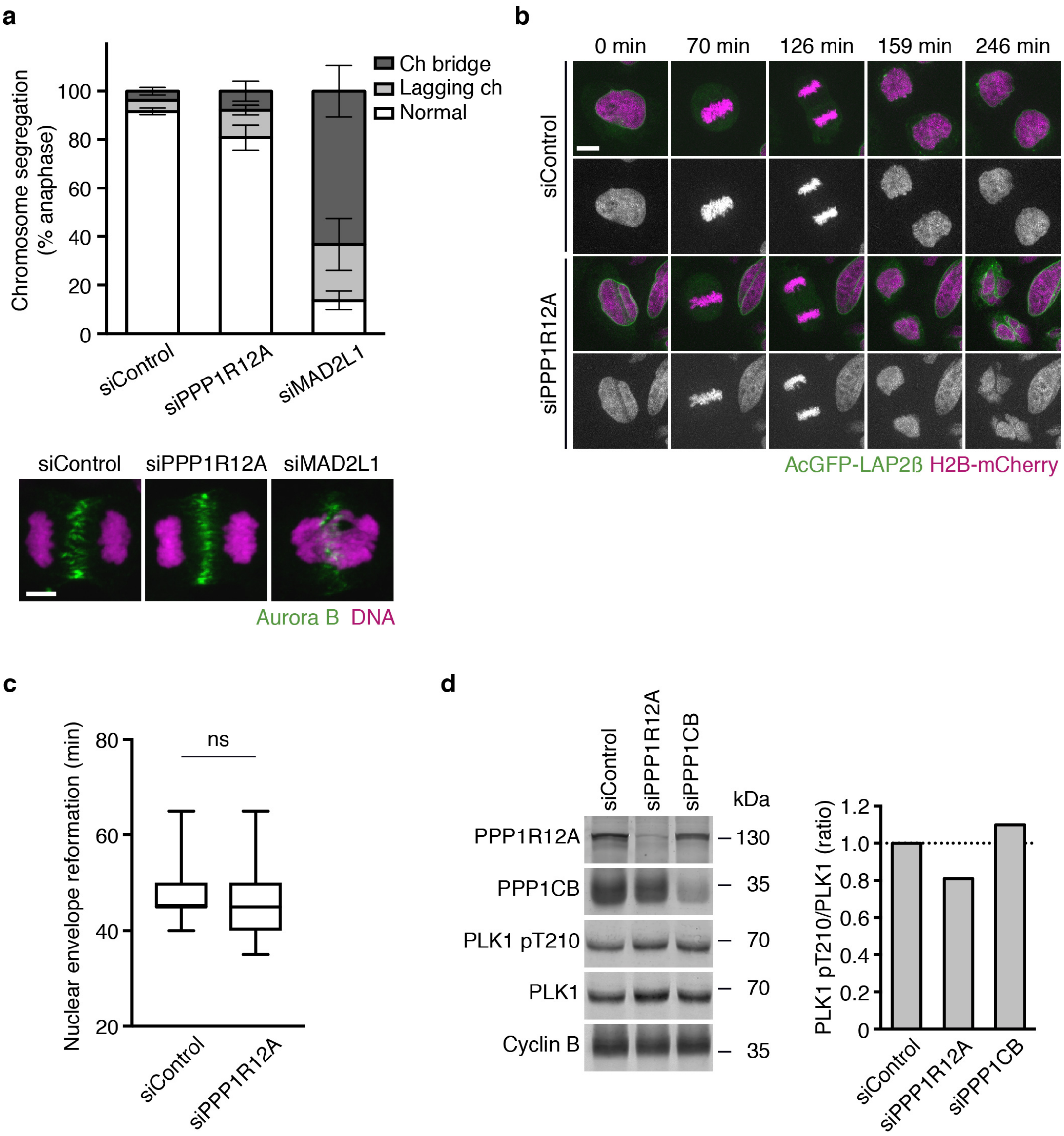

Supplementary Figure 6 | Effect of PPP1R12A depletion on mitosis and nuclear envelope reformation.
(a) Quantification of chromosome segregation in anaphase cells transfected with the indicated siRNAs (top panel). Error bars, s.d. of four independent experiments (n > 20 anaphase cells each). Representative image of anaphase cells treated with the indicated siRNA duplexes (bottom panel). **(b)** Time-lapse analysis of mitosis and mitotic exit in AcGFP-LAP2ß and H2B-mCherry expressing HeLa cells that were transfected with the indicated siRNA duplexes. Cells were recorded from 50 h after siRNA transfection onwards in intervals of 3 min. **(c)** Timing of nuclear envelope reformation after anaphase onset in control and PPP1R12A siRNA-transfected cells (see methods section for details). (siControl; n = 19, siPPP1R12A; n = 21 cells each). Graph shows ns; non-significant (unpaired t test). **(d)** Effect of PPP1R12A and PPP1CB depletion on PLK1 T210 phosphorylation. Cells were transfected with the indicated siRNA duplexes. 50 h after transfection cells were treated with 100 ng/ml nocodazole for 6 h. Mitotic cells were analyzed by quantitative immunoblotting (left panel). The ratio of total PLK1 and PLK1 phospho-T210 was normalized to the ratio observed in siControl-transfected cells and plotted (right panel). Scale bars, 10 µm (a and b).

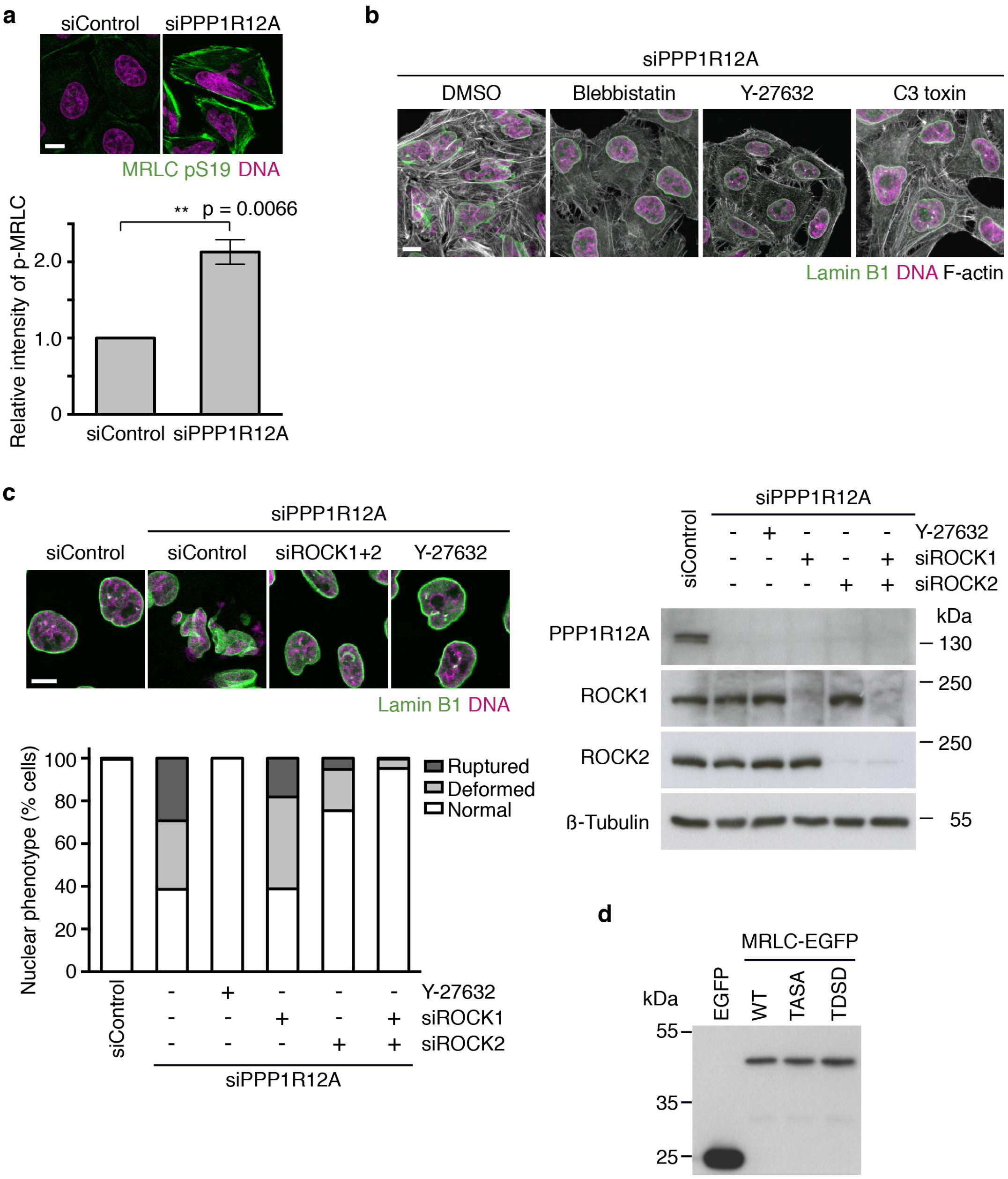

Supplementary Figure 7 | Actomyosin contractility drives nuclear deformation and rupture. (a) Quantification of phospho-MRLC. HeLa cells were transfected with PPP1R12A siRNA and fixed 56 h after transfection. Representative images of phospho-S19 MRLC detection (top panel). The mean cellular intensity of phosphoS19-MRLC was calculated and normalized to cells transfected with siControl (bottom panel). Error bars, s.d. of three independent experiments (n > 100 cells each). P values, unpaired t test. **(b)** Representative immunofluorescence images of PPP1R12A siRNA-transfected cells that were treated with 5 μM blebbistatin, 5 μM Y-27632, or 0.4 μg/ml C3 toxin for 24 h before fixation. **(c)** Suppression of nuclear deformation by ROCK depletion or inhibition. HeLa cells were transfected with indicated siRNA duplexes. 56 h after transfection nuclear morphology was analyzed by immunofluorescence. PPP1R12A-depleted cells were treated with Y-27632 as indicated. Representative images (upper panel) and quantification of nuclear phenotype (lower panel) (n > 200 cells). Protein depletion was analyzed by immunoblotting (right panel). **(d)** Immunoblot detection of MRLC-EGFP transgene expression in HeLa cells 72 h after infection with lentiviral particles. Scale bars, 10 μm (a, b, and c).

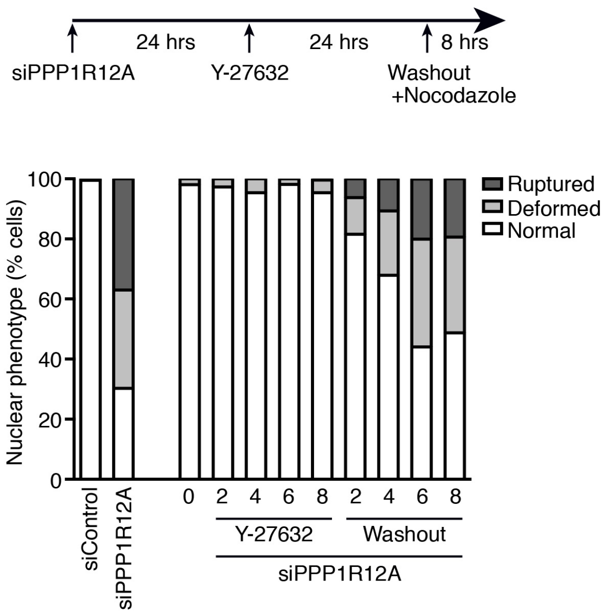

Supplementary Figure 8 | Nuclear deformation without passage through mitosis. HeLa cells were transfected with PPP1R12A siRNA. Y-27632 was added 24 h after transfection. Y-27632 was washed out after 24 h and cells were treated with 50 ng/ml nocodozole to prevent exit from mitosis. Cells were fixed and nuclear morphology analyzed at the indicated time points after Y-27632 washout (n > 200 cells).

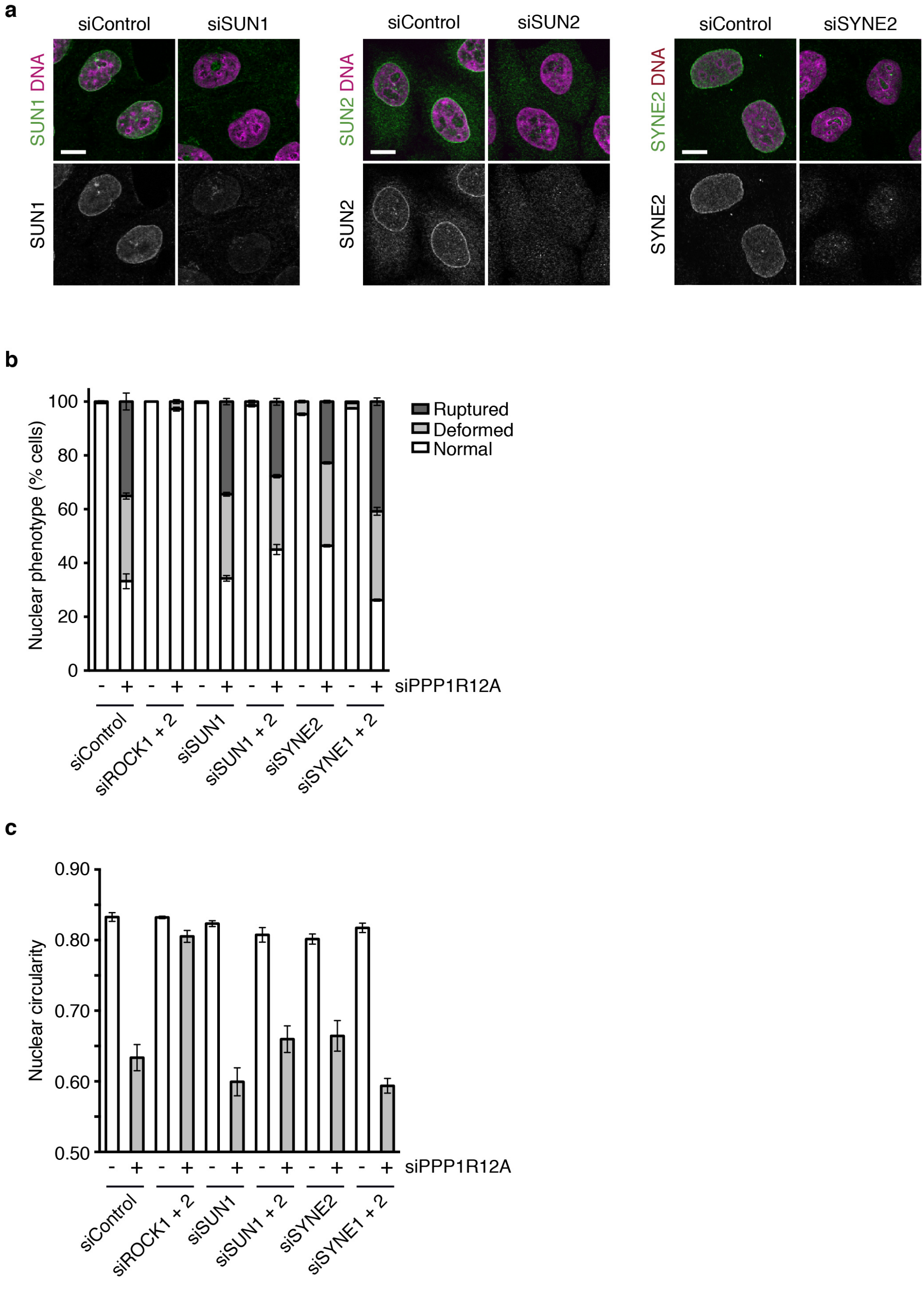

Supplementary Figure 9 | The role of LINC complex proteins. **(a)** Representative immunofluorescence images of cells transfected with siControl or siRNA duplexes targeting SUN1, SUN2, and SYNE2. Scale bars, 10 µm. **(b)** Quantification of the nuclear phenotype in cells treated with the indicated siRNAs. Error bars, s.d. of three independent experiments (n > 200 cells). **(c)** Nuclear circularity of cells treated with the indicated siRNAs. Error bars, s.d. of three independent experiments (n > 100 nuclei each).

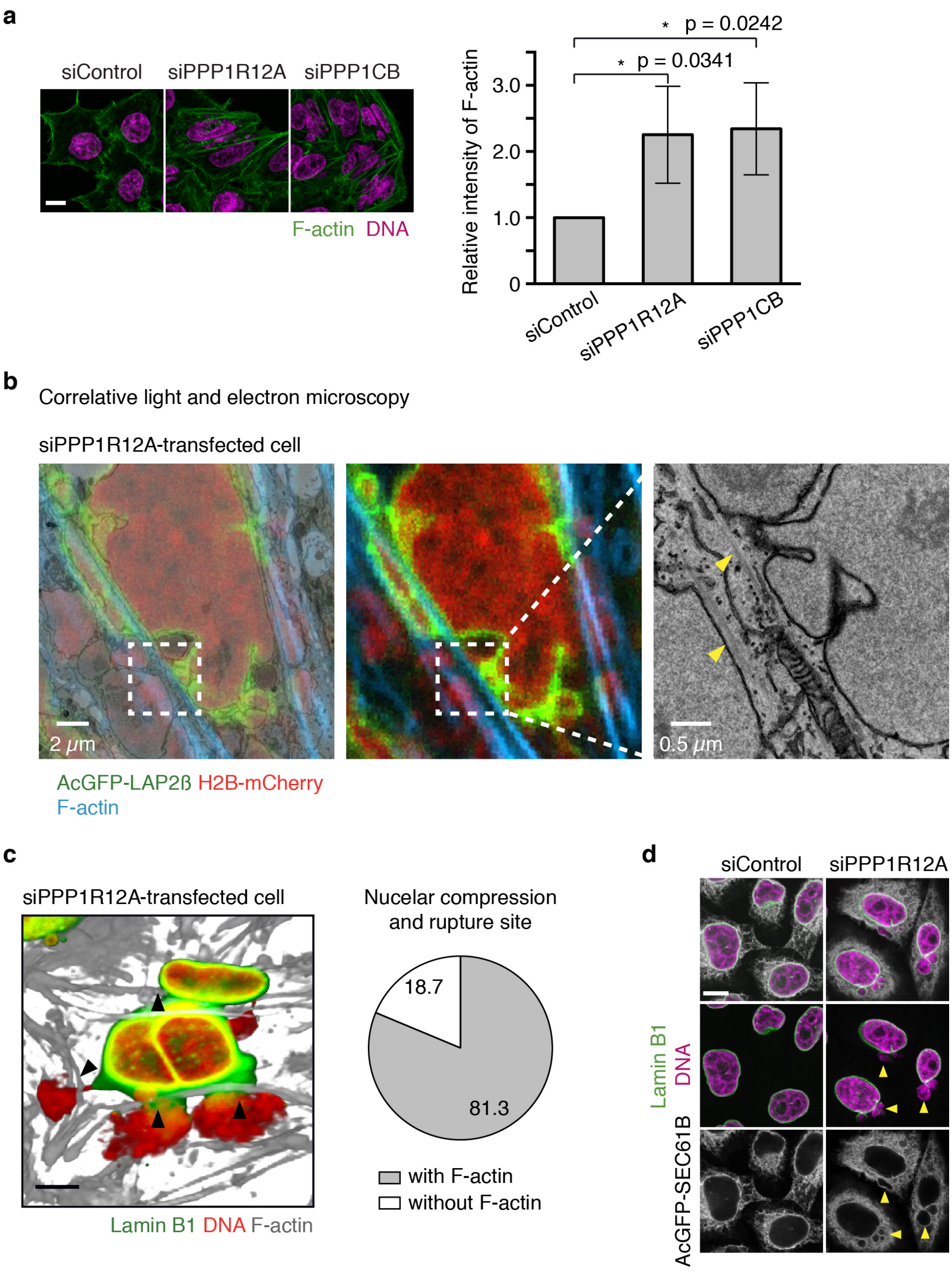

Supplementary Figure 10 | Analysis of nuclear envelope rupture sites. (a) Quantification of filamentous actin (F-actin) in PPP1R12A and PPP1CB-depleted cells. Representative images of HeLa cells transfected with the indicated siRNAs, fixed and stained with fluorophore-conjugated phalloidin and DAPI (left panel). The mean cellular intensity of F-actin was calculated by subtracting the mean value of F-actin intensity in cells treated with 5 μM latrunculin A. Subsequently, resulting values were normalized to the value measured in siControl transfected cells (right panel). Error bars, s.d. of three independent experiments (n = 100 cells each). P values, one-way ANOVA Tukey's multiple comparison test. **(b)** Correlative light and electron microscopy analysis of HeLa cells stably expressing AcGFP-LAP2ß and H2B-mCherry that were transfected with PPP1R12A siRNA for 56 h. Cells were exposed to 2 μM SiR-actin for 1 h before fixation. Overlay of EM and fluorescence image (left panel), fluorescence image (middle panel), and high magnification EM image (right panel). The dashed rectangles indicate the area shown in the high magnification EM image. Yellow arrowheads indicate fiber structures. Scale bars, as indicated. **(c)** PPP1R12A-depleted cells were fixed and stained for with anti-lamin B1 antibodies, DAPI and fluorophore-conjugated phalloidin. Cells were scanned in 0.1 μm sections using confocal laser scanning microscope. The 3D image was reconstituted and rendered using Imaris software. Arrowheads indicate F-actin bundles associated with nuclear compression and rupture sites. Quantification of nuclear compression and rupture sites associated with F-actin bundles (right panel) (n = 203). **(d)** Representative images of HeLa Kyoto cells that were transfected with control or PPP1R12A siRNA and with a plasmid encoding an AcGFP-SEC61B transgene. Arrowheads indicate DNA areas that are not covered by lamin B1 but that are associated with AcGFP-Sec61B. Scale bar, 10 μm (a, c, and d).

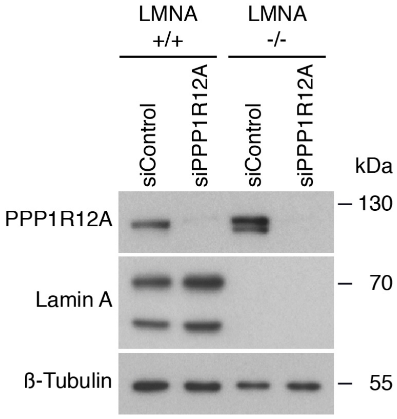

Supplementary Figure 11 | Analysis of *LMNA-/-* MEFs. Immortalized *LMNA+/+* and *LMNA-/-* mouse embryonic fibroblasts were transfected with control or PPP1R12A siRNA duplexes and analyzed by immunoblotting.

a

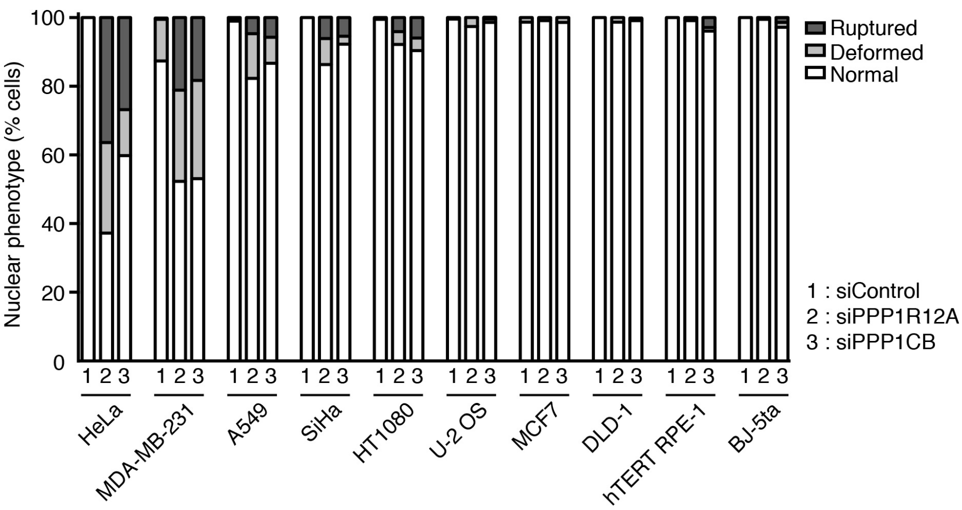

b

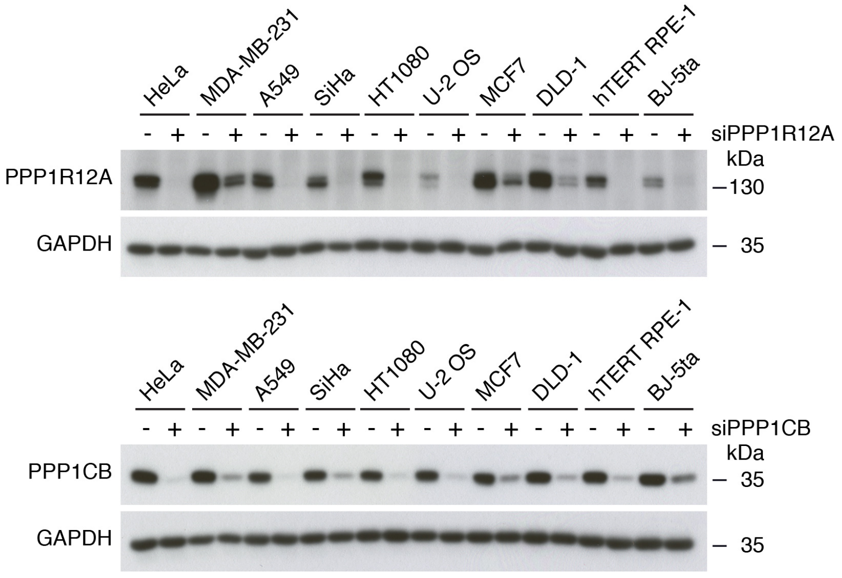

Supplementary Figure 12 | Effect of PPP1R12A and PPP1CB depletion in a panel of cell lines. (a) Quantification of nuclear morphology in a panel of cell lines after transfection with the indicated siRNA duplexes (n > 200 cells). Transfected cells were fixed 56 h after transfection and stained with anti-lamin B1 antibodies and DAPI. **(b)** Immunoblot analysis of protein depletion.

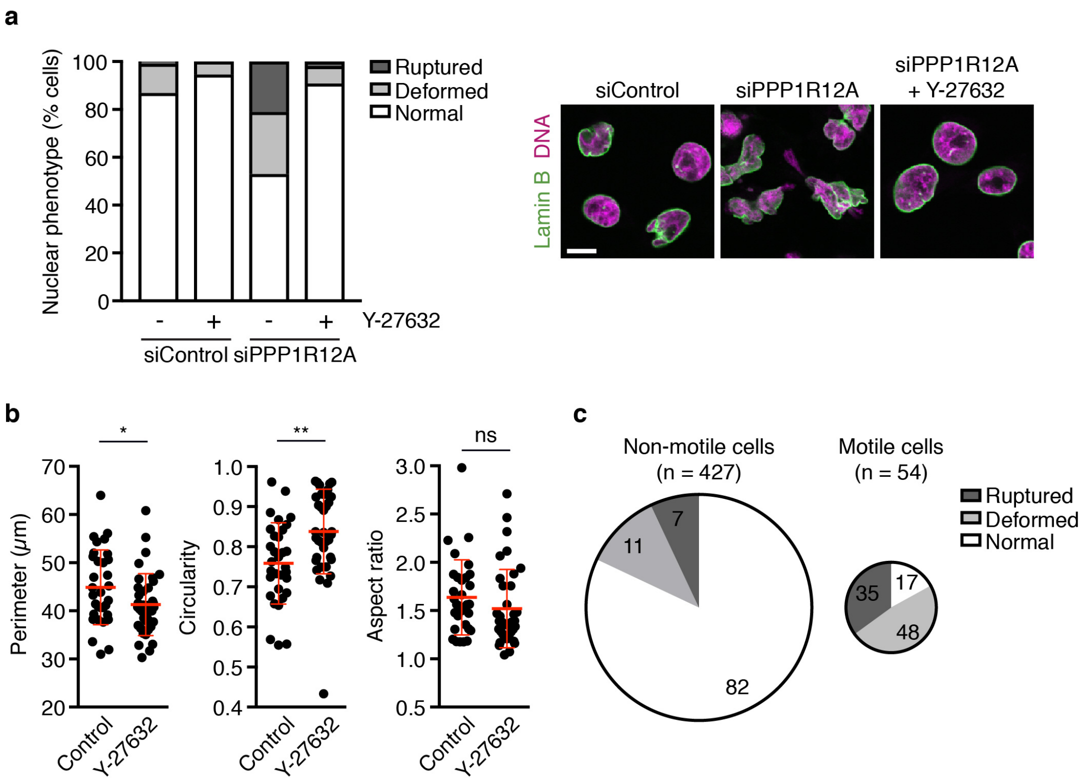

Supplementary Figure 13 | Analysis of nuclear morphology in MDA-MB-231 cells *in vitro* and *in vivo*.
(a) Quantification of nuclear phenotype caused by PPP1R12A depletion and treatment with Y-27632 in MDA-MB-231 cells (left panel). Representative cell images of MDA-MB-231 cells (right panel). (n > 200 cells). Scale bar, 10 μm. **(b)** Nuclear perimeter, circularity, and aspect ratio of individual MDA-MB-231 cells within an untreated (water) xenografts or within an Y-27632-treated xenograft were calculated and plotted (control; n = 32, Y-27632; n = 37 cells each). Graph shows *P = 0.0392, **P = 0.0022, ns non-significant (unpaired t test). **(c)** Comparison of nuclear deformation in motile and non-motile cells as observed using intravital imaging of MDA-MB-231 cells stably expressing AcGFP-LAP2β and H2B-mCherry. The area of the charts reflects the number of non-motile and motile cells in the movies analysed (n = 427 and 54 cells), respectively.

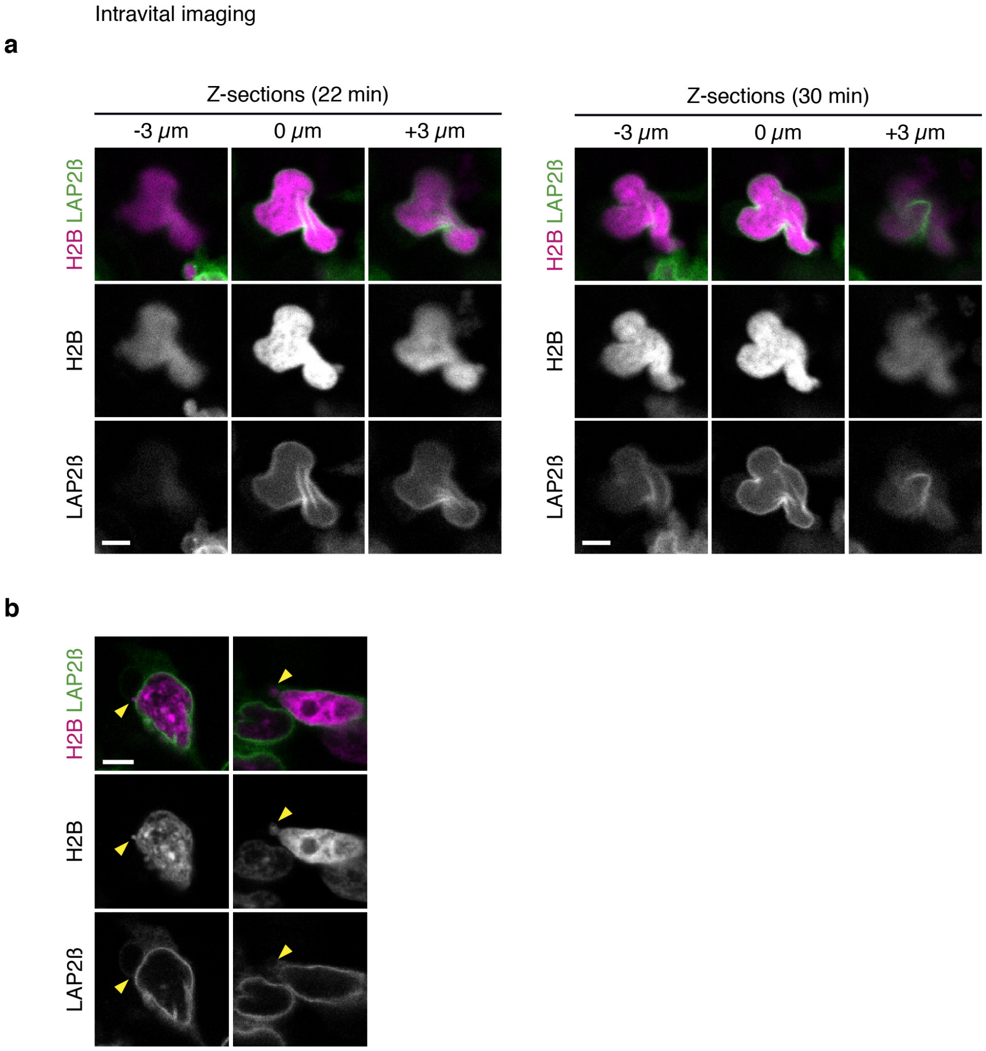

Supplementary Figure 14 | Additional images and serial z-sections of intravital time-lapse analyses. (a) Serial z-stack images of the intravital time-lapse series of an MDA-MB-231 tumor expressing AcGFP-LAP2β and H2B-mCherry shown in Fig. 5c. **(b)** Intravital cell images of an MDA-MB-231 tumor expressing AcGFP-LAP2β and H2B-mCherry recorded with a 63x 1.2NA objective. The arrowheads indicate nuclear envelope rupture events. Scale bars, 10 µm.

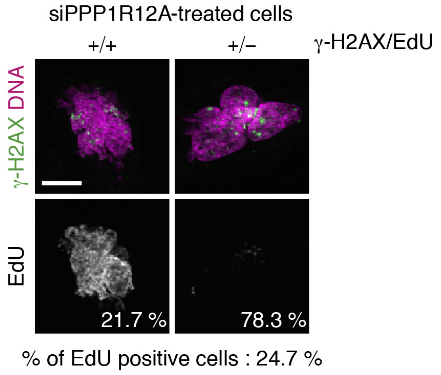

Supplementary Figure 15 | DNA damage caused by PPP1R12A depletion in replicating and non-replicating cells. HeLa Kyoto cells were transfected with PPP1R12A siRNA and labelled with EdU for 2h before fixation. Cells were stained for EdU, ɣ-H2AX, and DNA. Representative images of EdU-positive (left) and EdU-negative (right) cells containing a ɣ-H2AX signal. The percentages of ɣ-H2AX-positive cells that are EdU-positive and EdU-negative are shown above the images. The percentage of EdU-positive cells in the population irrespective of ɣ-H2AX status is shown below the images.

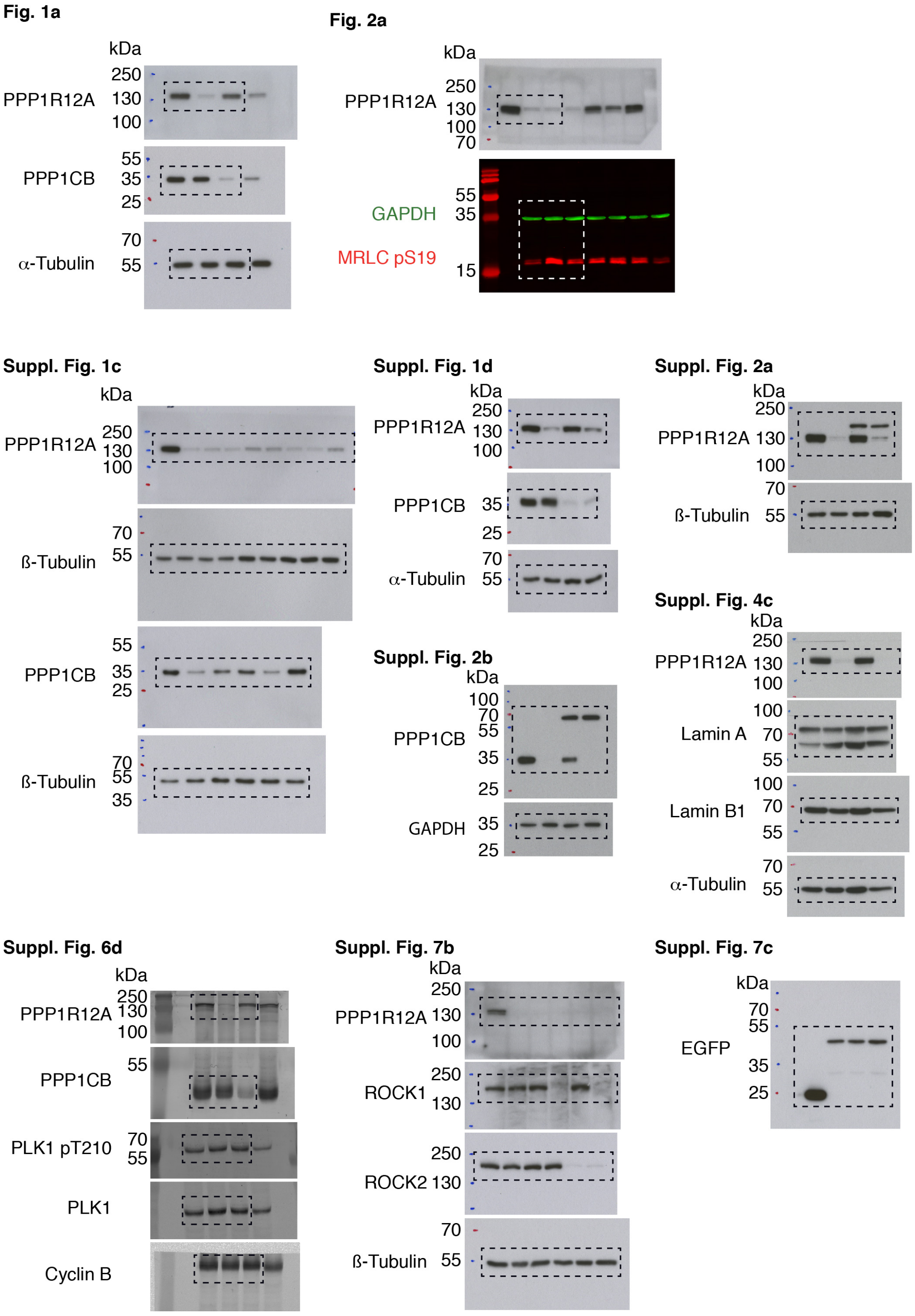

Supplementary Figure 16 | Full-size versions of immunoblots shown in main and Supplementary Figures. See next page for Supplementary Figure 16 continued.

Suppl. Fig. 11

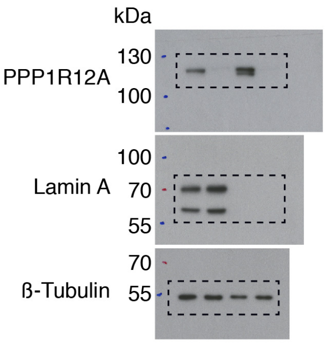

Suppl. Fig. 12

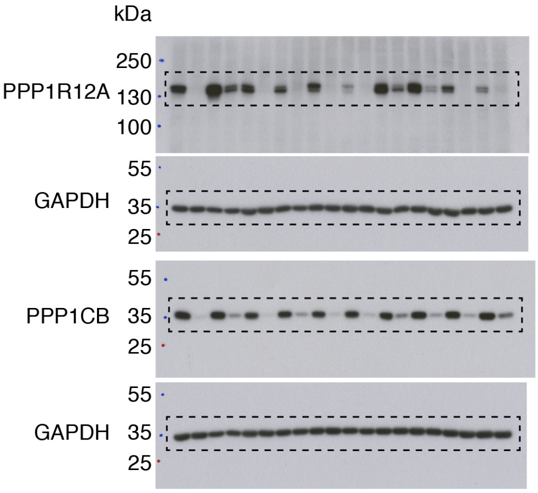

Supplementary Figure 16 continued.

Antigen	Supplier	Catalogue number	IF dilution	IB dilution
PPP1R12A	Santa Cruz Biotech	sc-25618		1:500
PPP1CB	Abcam	ab53315		1:50000
Lamin A	Abcam	ab8980	1:1000	
Lamin A	Cell Signalling Tech	4777		1:2000
Lamin B1	Abcam	ab16048	1:1000	1:5000
LAP2ß	BD Biosciences	611000	1:1000	
NPC	Abcam	ab50008	1:500	
SUN1	Novus Biologicals	NBP1-87396	1:400	
SUN2	Abcam	ab124916	1:100	
SYNE2	Thermo Scientific	K20-478-5	1:1000	
PML	Abcam	ab96051	1:500	
ROCK1	BD Biosciences	611137		1:250
ROCK2	Santa Cruz Biotech	sc-5561		1:1000
PLK1	Santa Cruz Biotech	sc-17783		1:2000
PLK1 pT210	Cell Signalling Tech	5472		1:1000
Cyclin B	Santa Cruz Biotech	sc-245		1:2000
MRLC pS19	Cell Signalling Tech	3671	1:50	1:1000
Aurora B	BD Biosciences	611082		1:500
γ-H2AX	Millipore	05-636	1:600	
Cytochrome c	Life Technology	33-8200	1:25	
GFP	Roche	118144600001	1:1000	1:1000
GAPDH	Abcam	ab8245		1:40000
AcGFP	Clontech	632381	1:1000	
53BP1	Novus Biologicals	NB100-305	1:1200	
Cleaved PARP	Cell Signalling Tech	5625	1:400	
α-tubulin	Sigma	T6074		1:20000
ß-tubulin	Cell Signalling Tech	5346		1:2000
Phalloidin Alexa488	Life Technology	A12379	1:500	
Phalloidin Alexa568	Life Technology	A12380	1:500	

Supplementary Table 1 | Antibodies used in this study. Antigen, supplier, catalogue number, dilution for immunofluorescence (IF) staining and dilution for immunoblotting (IB) are listed.

Target (siRNA #)	Species	Supplier	Catalogue number
PPP1R12A pool	human	Dharmacon	M-011340-01
PPP1R12A-1	human	Dharmacon	D-011340-02
PPP1R12A-2	human	Dharmacon	D-011340-04
PPP1R12A-3	human	Dharmacon	D-001340-05
PPP1R12A-4	human	Dharmacon	D-011340-06
PPP1R12A-5	human	Invitrogen	HSS106921
PPP1R12A-6	human	Invitrogen	HSS106922
PPP1R12A-7	human	Invitrogen	HSS106923
PPP1R12A	mouse	Dharmacon	D-063177-04
PPP1CB pool	human	Dharmacon	M-008685-00
PPP1CB-1	human	Dharmacon	D-008685-01
PPP1CB-2	human	Dharmacon	D-008685-02
PPP1CB-3	human	Dharmacon	D-008685-03
PPP1CB-4	human	Dharmacon	D-008685-04
ROCK1	human	Dharmacon	M-003536-02
ROCK2	human	Dharmacon	M-004610-02
MAD2L1	human	Invitrogen	HSS106245
SUN1	human	Dharmacon	M-025277-01
SUN2	human	Dharmacon	M-009959-00
SYNE1	human	Dharmacon	M-014039-01
SYNE2	human	Dharmacon	M-019259-01
Control		Dharmacon	D-001210-01

Supplementary Table 2 | siRNA duplexes used in this study. Targets, species, supplier and catalogue number are listed.

Name	Supplier	Catalogue number
Y-27632	Sigma	Y0503
Blebbisitatin	Sigma	N0560
C3 toxin	Cytoskelton	CT04
Aphidicolin	Sigma	A0781
Actinomycin D	Sigma	A9415
DAPI	Life Technology	D21490
Caffeine	Sigma	C0750
ML-7	Sigma	I2764
DPX mountant	Sigma	44581
Giemsa	Sigma	48900
SiR-actin	Spirochrome	SC001
Nocodazole	Sigma	M1404
MG132	Sigma	C2211
Thymidine	Sigma	T1895
Click-iT Plus EdU Imaging Kit	Life Technology	C10639

Supplementary Table 3 | Chemicals and reagents used in this study. Name, supplier and catalogue number are listed.
